# Genetic and physical mapping of loci for resistance to blackleg disease in canola (*Brassica napus* L.)

**DOI:** 10.1038/s41598-020-61211-y

**Published:** 2020-03-10

**Authors:** Rosy Raman, Simon Diffey, Denise M. Barbulescu, Neil Coombes, David Luckett, Phil Salisbury, Raymond Cowley, Steve Marcroft, Harsh Raman

**Affiliations:** 1 NSW Department of Primary Industries, Wagga Wagga Agricultural Institute, Wagga Wagga, NSW 2650 Australia; 2 Apex Biometry, South Freemantle, Perth, WA 6162 Australia; 3 Agriculture Victoria, Grains Innovation Park, Horsham, 3401 VIC Australia; 4 Graham Centre for Agricultural Innovation (a joint venture between Charles Sturt University and NSW Department of Primary Industries), Charles Sturt University, Boorooma Street, Wagga Wagga, NSW 2650 Australia; 5 Faculty of Veterinary and Agricultural Sciences, The University of Melbourne, Vic, 3010 Australia; 6 Present Address: Corteva Agroscience, Lawson Street, Wagga Wagga, NSW 2650 Australia; 7 Marcroft Grains Pathology, Grains Innovation Park, Horsham, Victoria 3400 Australia

**Keywords:** Plant sciences, Plant sciences, Plant breeding, Plant breeding

## Abstract

Sustainable canola production is essential to meet growing human demands for vegetable oil, biodiesel, and meal for stock feed markets. Blackleg, caused by the fungal pathogen, *Leptosphaeria maculans* is a devastating disease that can lead to significant yield loss in many canola production regions worldwide. Breakdown of race-specific resistance to *L. maculans* in commercial cultivars poses a constant threat to the canola industry. To identify new alleles, especially for quantitative resistance (QR), we analyzed 177 doubled haploid (DH) lines derived from an RP04/Ag-Outback cross. DH lines were evaluated for QR under field conditions in three experiments conducted at Wagga Wagga (2013, 2014) and Lake Green (2015), and under shade house conditions using the ‘ascospore shower’ test. DH lines were also characterized for qualitative *R* gene-mediated resistance via cotyledon tests with two differential single spore isolates, IBCN17 and IBCN76, under glasshouse conditions. Based on 18,851 DArTseq markers, a linkage map representing 2,019 unique marker bins was constructed and then utilized for QTL detection. Marker regression analysis identified 22 significant marker associations for resistance, allowing identification of two race-specific resistance *R* genes, *Rlm3* and *Rlm4*, and 21 marker associations for QR loci. At least three SNP associations for QR were repeatedly detected on chromosomes A03, A07 and C04 across phenotyping environments. Physical mapping of markers linked with these consistent QR loci on the *B. napus* genome assembly revealed their localization in close proximity of the candidate genes of *B. napus* BnaA03g26760D (A03), BnaA07g20240D (A07) and BnaC04g02040D (C04). Annotation of these candidate genes revealed their association with protein kinase and jumonji proteins implicated in defense resistance. Both *Rlm3* and *Rlm4* genes identified in this DH population did not show any association with resistance loci detected under either field and/or shade house conditions (ascospore shower) suggesting that both genes are ineffective in conferring resistance to *L. maculans* in Australian field conditions. Taken together, our study identified sequence-based molecular markers for dissecting *R* and QR loci to *L. maculans* in a canola DH population from the RP04/Ag-Outback cross.

## Introduction

Blackleg, caused by the fungal pathogen *Leptosphaeria maculans*, is a major disease of the canola (*Brassica napus* L., A_n_A_n_C_n_C_n_, 2n = 4× = 38) industry especially in Australia, Canada, and France. Developing new cultivars resistant to blackleg is recognized as the most economically and environmentally safe strategy to control yield losses. Traditional breeding for blackleg resistance largely relies on the selection of elite ‘good looking’ lines in disease nurseries where there is high disease pressure from a diverse pathogen population. Utilizing this strategy, several canola varieties have been developed and released for commercial cultivation with ratings of moderate- to highly-resistant (to blackleg) without any prior knowledge of either race-specific resistance (qualitative resistance; *R*) and/or race non-specific resistance (quantitative resistance; QR) genes.

*R* gene mediated resistance generally follows the classical gene-to-gene hypothesis; although genetic interactions between *R* genes have been reported in *B. napus* - *L. maculans* pathosystem^1,2^. *R* genes often have high heritability due to the major allelic effects, which can be tested efficiently and accurately at the cotyledon stage^3,4^. When those resistant genotypes harboring *R* gene(s) are subsequently grown they also exhibit a resistance response at the adult plant stage under controlled conditions^3–5^. However, a consistent resistance response may not be detected when plants are grown under natural field conditions, as those individuals are constantly challenged with in-coming inoculum from sexually reproducing diverse races of *L. maculans* throughout the Australian canola growing season (March-November). Conversely, QR is usually controlled by several genes of smaller allelic effect however these QR alleles together confer more durable resistance at the adult plant stage under field conditions. Due to strong genotype × environment interactions, the effects of QR loci detected in the mapping populations are often relatively small compared to *R* genes^3,5–11^. Currently, deployment of QR is preferred by canola breeding programs due to its durability and lower chance of breakdown of resistance to the highly-diverse nature of *L. maculans* as observed in some Australian and French cultivars^12–14^.

Quantitative resistance has been genetically mapped in a relatively limited number of structured bi-parental doubled haploid (DH) populations of canola using QTL linkage mapping^3,4,8,10^, and by genome-wide association approaches^5,15–17^. These studies showed that (i) multiple QTL are located on all 19 chromosomes of *B. napus* which control resistance to *L. maculans*, and (ii) QTL allelic effects vary depending upon the environment and the method of evaluation (per cent survival or internal infection scores). In addition, due to the usage of different genetic populations with spring, semi-winter, or winter backgrounds plus a range of molecular marker systems, it has been difficult to compare the locations of QTL for QR in canola from different studies. Recently, the genome of *B. napus* cv. Darmor-*bzh*, along with the physical positions of 425 *R* genes implicated in disease resistance, has been published^18,19^. This valuable resource provides an opportunity to compare QTL regions involved in resistance to *L. maculans* across populations, and to identify putative candidate genes underlying *R* and QR loci.

In this study, we identified loci associated with *R* and *QR* using linkage analysis and located their physical map positions on the Darmor genome assembly enabling the identification of putative candidate genes. Comparisons were also made between the significant QTL detected in the present study with those from previous studies which utilized sequence-based markers.

## Materials and methods

### Plant materials and single spore isolates

A DH population (RADH) comprising 177 lines was developed from a F_1_ cross between two Australian genotypes RP04 and Ag-Outback using microspore culture. This breeding DH population was generated at the Agriculture Victoria, Grains Innovation Park, Horsham, Australia. RP04 is an advanced breeding line developed by Agriculture Victoria (Ag-Vic) while Ag-Outback is a commercial canola variety developed from the cross: AGA95-1/Monty by AgSeed Pty Ltd (now Nuseed Pty Ltd, Australia) and released for cultivation in Australia^20^. Both parental genotypes were moderately resistant to blackleg disease under field conditions (Salisbury, unpublished data) and also shown to differ in fractional ground cover^21^, flowering time, plant height and seed yield (Raman *et al*., unpublished data). Seed of DH lines was multiplied in caged tents to avoid any cross pollination. A set of 10 *L. maculans* differential isolates^22^ was procured from Marcroft Grains Pathology, Horsham and maintained on 10% V8 juice agar (Campbell, Australia) at the Wagga Wagga Agricultural Institute (WWAI) for phenotyping experiments.

### Phenotypic evaluation of DH lines

#### Evaluation of RADH population for resistance under field conditions

The RADH population, along with its parental lines, RP04 and Ag-Outback, were evaluated under field conditions in three experiments during the canola cropping seasons in Australia (April to November); two experiments: experiment-I (rainfed, 35 °03′36.9″S 147 °18′40.2″E), experiment-II (irrigated, 35 °02′27.0″S 147 °19′12.6″E) in 2013 at the WWAI, and one experiment: experiment-III (rainfed, 36.773949, 142.273688) at Green Lake, Victoria in 2015.

For the field experiments at Wagga Wagga, 169 DH lines along with the parental lines, and 12 commercial varieties of *B. napus* as checks [ATR-Marlin, ATR-Stingray, AV-Garnet, CB-Tanami, CB-Telfer, CB-Trigold, Crusher-TT, Hurricane-TT, Hyola50, Hyola76, Tawriffic-TT and one accession of *Brassica juncea* (Dune)] were grown as single rows (10 m long) in field blackleg screening nurseries, and spaced 80 cm apart in a randomised complete block design with three replications. The rows were trimmed to 8 m at growth stage 50 when plants reached the rosette stage. The nursery was planted on previous canola stubble that had been sprayed-out with glyphosate prior to seed set. The plants in the preceding stubble crop were a mixture of various genotypes with different known *R* genes in order to provide a diverse pathogen population for the subsequent season. The experiment-II trial was flood-irrigated (approximately 50 mm) once in September 2013 at the stem elongation stage to promote disease development. Trials were conducted as per standard agronomic practice, fertilized with 150 kg N, and sprayed with appropriate herbicides to control weeds when required.

For the experiment-III trial at Green Lake, 165 DH lines of RADH along with RP04, Ag-Outback and 22 *B. napus* commercial cultivars (44C79, 44Y84CL, 45Y86CL, ATR-Gem, ATR-Marlin, ATR-Stingray, CB-Sturt HT, CB-Telfer, Crusher TT, Hyola444TT, Hyola-50, Hyola 559TT, Hyola 575CL, JBOT800407, Nuseed Diamond, Pioneer 44Y26, Pioneer 44Y89, SturtTT, ThumperTT, Trigold, Victory V3002, and Westar) and two *B. juncea* cv. Dune and OasisCL as checks were sown *in situ* in the previous year’s canola stubble of cultivar ATR-GEM (*Rlm1*) on 28 May 2015. The trial was designed in AGROBASE using the ‘Grid Nursery with Checks’ model, which is a complete randomised block, with single row and two replicate design, and has a check *cv*. Trigold inserted every 10th row throughout the trial. The data collected from the disease nursery were: emergence counts at 6 weeks after sowing, adult plant survival counts and internal infection levels, both at physiological maturity. Up to 20 randomly selected plants from each row were cut from the crowns and scored for percent area discoloration of the stem due to blackleg fungus growth, as per scale described previously^22^.

#### Evaluation for resistance with ascospore shower test

171 DH lines from RADH and the parental lines, along with two controls; Skipton and Ag-Spectrum, were evaluated by Ag-Vic/Marcroft Grains Pathology for resistance to *L. maculans* pathotypes released from a mixed stubble using the ascopsore shower test^23^. Canola stubble was collected from commercial crops of AV-Garnet (MT Hope, South Australia), CB-Jardhee HT (Frances, South Australia), Monola76TT (Bool Lagoon, South Australia) and ATR-Cobbler (Wagga Wagga, NSW), grown in the 2011 cropping season. Details of the ‘ascospore shower’ test are given in Marcroft *et al*.^22^. This experiment was conducted in plastic pots (20 cm diameter) under glasshouse conditions in 2013 using a multiphase design comprising (i) an inoculation chamber phase and (ii) a glasshouse phase in order to account for non-genetic sources of variation in both phases. All entries after inoculation were laid in two chambers as shown in Supplementary Fig. 1. Each inoculation chamber accommodated a complete replicate of 200 entries (171 RADH lines, 2 entries of each parental lines; RP04 and Ag-Outback and 13 entries of Skipton and 12 entries of Ag-Spectrum) plus 50% of lines (100 lines) representing the third replicate. Each chamber had a complete array of 20 rows by 30 columns, ensuring that no two DH lines would be located next to each other across replicates. Four plants of each genotype were used as the experimental unit with each plant being scored. Spatial effects were modelled separately for both chambers. The design was generated using optimal design (od) software^24^ in R. At the physiological maturity stage (GS 83–85), the inoculated plants were cut at the crowns and cross sections were assessed visually for disease severity as described previously^3^.

#### Evaluation of RADH population for *R* genes

To determine whether *R* genes that confer resistance to *L. maculans* under controlled glasshouse conditions are the same with the ones contributing towards QR detected under field conditions, parental lines, RP04 and Ag-outback and their DH lines were characterized for resistance to *L. maculans* with 10 single-spore isolates (SSI) via cotyledon tests. Two isolates, IBCN17-D4 (*AvrLm4, AvrLm5, AvrLm6, AvrLm7, AvrLm8, AvrLepR3, AvrLepR1, AvrLepR4*) and IBCN76-D7 (*AvrLm1, AvrLm3, AvrLm5, AvrLm6, AvrLm8, AvrLepR3, AvrLepR1, AvrLepR4*)), that showed differential disease reactions on the parental lines (Supplementary Table 1a) were used for the genetic analysis of 177 DH lines of RADH in two independent experiments.

A randomized complete block design was generated using the spatial design search program DiGGer (Coombes 2002) assuming positive correlation between neighboring cells in rows and columns and allowing for random row and column effects within a column-pair of benches and further used for evaluating the DH population. A total of 171 DH lines, two parental lines and nine control cultivars; Westar (no *R* gene), AV-Garnet (*Rlm1*, 9), Q2 (*Rlm3*,*9*), Thunder TT (*Rlm4*), Caiman (*Rlm7*), Surpass400 (*Rlm1* and *LepR3*), Hyola50 (*Rlm1*, *LepR1*), Mustang (*Rlm6*) and 44C79C (*Rlm* unknown) were evaluated in two replicates across two benches, each bench had a replicate of 23 trays, with each tray containing a row of 8 genotypes representing either DH and/or parental or control cultivars. The seedlings were grown in plastic trays (7 × 8 cells) in a controlled glasshouse at WWAI and maintained at 20 ± 2 °C for two weeks. Seven plants of each DH line (the experimental unit in a tray) were inoculated with the single spore isolate at a concentration of 10^6^ spores mL^−1^ as described previously^3^. The inoculated plants were then placed in a humidity chamber for 48 h at 100% relative humidity in the dark at 18 °C to allow spore germination. Subsequently, inoculated plants were transferred to the glasshouse and maintained at 20 ± 3 °C for 3–4 weeks. Disease symptoms on cotyledons were scored 21 days post-inoculation using a scale described previously^25^: 0 (resistant response/no disease development) to 9 (highly susceptible/and profuse sporulation). Quantitative disease scores were used to identify significant trait-marker associations.

### Phenotypic data analysis

**Figure 1:**
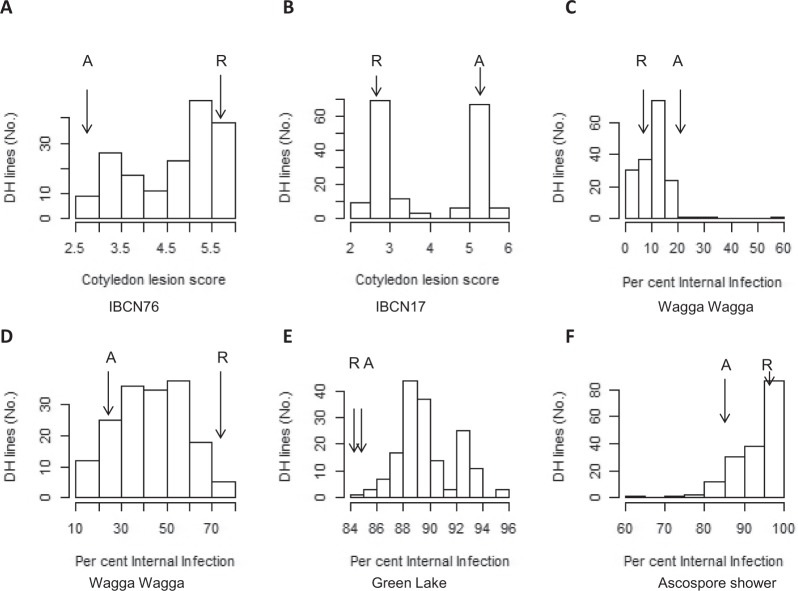
Distribution of disease scores in 177 doubled haploid lines from RP04/Ag-Outback evaluated for resistance to *L. maculans* under glasshouse using two single spore isolates; IBCN76 (**A**) and IBCN17 (**B**), field (**C**–**E**), and shade house conditions (‘ascospore shower’ test) (**F**). Disease severity was assessed as internal canker formation on a 0 to 100 scale (Raman *et al*. 2012). Disease lesions on cotyledons were measured 21 days post-inoculation. (**C**) Arrows indicate the mean disease scores of RP04 and Ag-Outback at 21 days post-inoculation.

Linear mixed model technology was used to fit a statistical model to phenotypic data generated by each experiment. The statistical model for each experiment included terms synonymous with a partitioning of the total variation into genetic and non-genetic sources of variation. Genetic sources of variation were further divided into variation due to DH lines and non-DH lines (which includes parental lines and commercial cultivars). Non-genetic sources of variation included terms associated with design factors associated with each experiment and in the case of field trials may also have included terms associated with the modelling of field spatial variability^26^. A logistic transformation was used for plant scores, internal infection percentage and survival percentage so that the statistical models for these traits better satisfied an assumption of normality and constant variance. The statistical model for each trait/experiment combination was used to form DH line Empirical Best Linear Unbiased Predictions (EBLUP’s) which are used in Fig. 1 to illustrate the range in resistance to *L. maculans;* and to estimate a broad sense heritability (*h*^2^*)*^27^ associated with DH lines. All statistical models were fitted using the statistical software package “asreml”^28^ within the R computing environment^29^.

### DArTseq genotyping

Genomic DNA was isolated from young leaves of 2–3 week-old seedlings of parental and DH lines from the RADH population using a standard phenol-chloroform method. The DNA samples were sent to Diversity Arrays Technology Pty. Ltd. (DArT P/L, University of Canberra, Bruce, Australia) for genotyping. Both parental lines and their DH lines were analysed using a DArTseq marker approach, as detailed in Raman *et al*.^30^. DArTseq markers were assigned to chromosomes based on their physical positions on the *B. napus* cv ‘Darmor-*bzh*’ genome assembly^19^.

### Linkage map construction and QTL analysis

DArTseq markers having <80% call rate, and <5% MAF were discarded prior to linkage analysis. A set of 18,851 DArTseq-SNPs and silico DArTs that showed segregation among DH lines was selected for genetic linkage map construction as described in^31^. QTL analysis involved taking the statistical models for phenotypic data and partitioning DH line genetic variation into variation due to putative QTL and polygenic variation. Putative QTL for resistance to blackleg disease were identified using a genome scan^31,32^ as well as the whole genome average interval mapping approaches^33^. In this study, we considered trait-marker associations as ‘significant’ having LOD scores ≥3 and ‘suggestive’ with LOD score ≥2 for all environments (experiments). However, researchers can make their own decisions on the usefulness of putative QTL when developing their own marker-assisted selection strategies. Trait-marker association was graphically represented using the Map Chart software^34^.

### Physical mapping and identification of putative candidate *R* genes

Sequences of markers significantly associated with resistance to blackleg were aligned to the reference *B. napus* cv. Darmor-*bzh* genome with the default BLAT settings^19^. Physical positions of *R* genes were downloaded from the public *B. napus* genome database (http://www.genoscope.cns.fr). Comparisons were made between the physical positions of *B napus* disease *R* genes and significant ‘hits’ using Microsoft Excel software. Candidate genes which were mapped within 200 kb of significant SNPs were checked for their annotations in TAIR (https://www.arabidopsis.org/) and *B. napus* genome databases. Biological functions of those genes in relation to *R* and QR loci were described.

## Results

### Inheritance of field resistance

**Figure 2:**
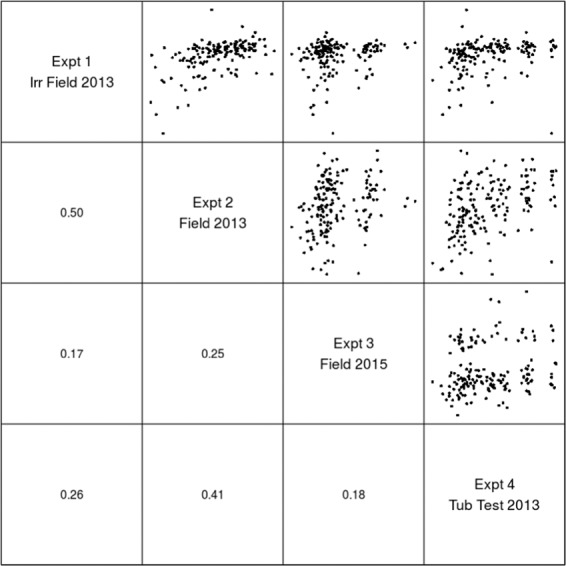
Pair-plots and raw Pearson genetic correlations of disease scores between doubled haploid lines (empirical best linear unbiased estimators) from the univariate analysis.

Significant genetic variation for resistance scored on the basis of internal canker formation was observed in field experiments (Table 1). The internal canker scores varied from 0.1 to 99.21% in the RADH population depending on the phenotyping environment (Table 1). Disease scores showed a continuous distribution for genetic variation in resistance among the DH lines of RADH grown under field conditions, suggesting that quantitative genes control resistance to *L. maculans* (Fig. 1). Internal canker scores were higher in the experiment at Green Lake compared to the Wagga Wagga site. Across different field experiments, canker scores showed moderate (44%) to high repeatability (broad sense heritability, *h*^2^ = 79%) except in the Green Lake experiment where *h*^2^ was low (12%). High *h*^2^ (61%) for resistance to *L. maculans* was observed among the DH lines evaluated with the ascospore shower test, suggesting that resistance was genetically controlled, and that this test is also suitable for identification of loci for resistance to blackleg (Table 1). The Pearson’s correlation coefficient analysis showed poor to moderate genetic correlation (*r* = 0.17 to 0.5) between disease scores across sites (Fig. 2). These results confirmed that phenotypic environment and/or specificity of host resistance to the diverse *L. maculans* population contributed significantly to the differential disease expression.

**Table 1:** Genetic variation and heritability for quantitative and qualitative resistance to *L. maculans* in a doubled haploid population from RP04/Ag-Outback.

Environment	Source of inoculum	Location	Number of DH tested	Broad sense heritability (h^2^%)	Disease score		
					RP04 (paternal parent)	Ag-outback (maternal parent)	Range in DH lines
**Internal infection assessed as crown canker (%)**							
Disease nursery (field-2013, Irrigated-site I)	Natural population of *L. maculans*	Wagga Wagga	169	79	24.91	9.88	0.09–58.19
Disease nursery (field-2013, Rainfed-site II)	Natural population of *L. maculans*	Wagga Wagga	169	44	53.42	26.0	10.26–75.86
Disease nursery (field-2015, Rainfed, site-III)	Natural population of *L. maculans*	Green Lake	165	12	38.22	58.43	84.65–95.58
Ascospore shower test (Screenhouse)	Mixed stubble for ascospore shower test	Horsham	171	61	95.76	89.65	63.74–99.92
**Cotyledon lesion score (0–9 scale)**							
Glasshouse	IBCN17 (D4)	Wagga Wagga	171	97	2.62	5.25	2.29–5.56
Glasshouse	IBCN76 (D7)	Wagga Wagga	171	85	5.65	2.66	2.60–5.80

Resistance to single spore isolates was scored as 0–9 (Koch *et al*. 1991). Internal infection was scored as 0 to 100% (Marcroft *et al*. 2012, Raman *et al*. 2012).

### Inheritance of resistance to single-spore isolates (SSI) of *L. maculans*

The parental lines of the RADH population were characterized for race-specific resistance using two SSI of *L. maculans*. Disease reactions showed that RP04 was resistant to the IBCN17 isolate, showing no visible critical disease symptoms (cotyledon score: 2.62) and Ag-Outback was susceptible (cotyledon lesion score: 5.26) (Supplementary Table 1b). However, Ag-Outback showed a resistance response to the IBCN76 isolate (2.66), while RP04 showed a susceptible response (5.65). Based on the differential response of parental lines of RADH population, Ag-Outback and RP04 are predicted to harbor *Rlm3* and *Rlm4* genes, respectively, for resistance to *L. maculans*. The distributions of cotyledon scores in the lines inoculated with IBCN17 and IBCN76 isolates showed a nearly bimodal distribution in a RADH population, suggesting that resistance to *L. maculans* could be due to race-specific *R* genes (Fig. 1A,B). The *h*^2^ estimates for disease scores were high, ranging from 85% to 97% for the IBCN76 and IBCN17 isolates, respectively (Table 1). The mean scores of DH lines ranged from 2.29 to 5.56 with IBCN17, compared to 2.60 to 5.80 with IBCN76. Significant negative correlation (r = −0.80) between disease scores of DH lines assessed with IBCN17 (*AvrLm4, AvrLm5, AvrLm6, AvrLm7, AvrLm8, AvrLepR3, AvrLepR1, AvrLepR4*) and IBCN76 (*AvrLm1, AvrLm3, AvrLm5, AvrLm6, AvrLm8, AvrLepR3, AvrLepR1, AvrLepR4*) was observed, suggesting that resistance in the population is contributed by different *R* genes (*Rlm3* and *Rlm4*) conferred by both parents having opposite allelic effects.

### Genetic mapping of RADH population

The RADH population was assayed with DArTseq-SNP and presence-absence (*in silico* DArTseq) marker variants. A total of 18,851 polymorphisms representing 6,442 SNPs and 12,409 *in silico* DArT markers were identified which showed segregation in the RADH population. Genotypic data of the RADH lines are presented in Supplementary Table 2. These polymorphic markers could be grouped into 2,019 unique bins, each one representing a single locus (Supplementary Table 3). Marker density on A subgenome was higher compared to C subgenome: 1051 bin markers were mapped on the subgenome A, covering 1,374.66 cM, and 968 bin markers were mapped on the subgenome C, covering 1,242.08 cM. The number of marker loci ranged from 149 on chromosome A08 to 1,942 on chromosome C04. On an average, bin marker density per cM ranged from 0.57 on chromosome A09 to 1.5 on chromosome C06, with a genome-wide coverage of 0.77 markers per cM. A framework linkage map based on 2, 019 bin markers (Supplementary Table 3) was further utilized for QTL analysis to identify trait-marker associations.

### Genetic analysis of DH population for resistance to blackleg

A total of 42 SNP marker associations (22 significant associations with LOD ≥ 3 and 20 suggestive associations with LOD ≥ 2) were identified using genome scan approach for resistance to *L. maculans* evaluated under (i) field disease nursery conditions, (ii) controlled conditions with SSI, and (iii) diverse pathotypes derived from mixed stubble using the ascospore shower test (Supplementary Table 4). The number of QTL identified ranged from 4 to 12 depending on the screening method/phenotyping environment/QTL detection method (Tables 2 and 3), and accounted for 1.2% to 90.1% of the observed genetic variation. Whole genome average interval mapping (WGAIM) identified 26 genomic regions (QTL) for resistance to *L. maculans* on the 13 chromosomes of *B. napus* (Table 3).

**Table 2:** Statistical association between DArTseq SNP markers and resistance to *L. maculans* in a doubled haploid population derived from the RP04/Ag-outback.

Phenotyping environment	DArTseq marker	Chromosome	Physical position of marker on the Darmor sequence	Probability of association	LOD Score	Genotypic variation explained (%)	Allelic effect
Field evaluation in disease nursery (Wagga Wagga)-site 1	5031260_16:C > A	A01	6158899	0.00	4.16	5.39	−0.29
	3118601_36:G > A	A06	20027453	0.00	3.16	4.83	0.20
	3091258_29:G > A	A10	11581532	0.00	3.18	5.14	0.22
Field evaluation in disease nursery (Wagga Wagga)-site II	**5238867_9:G** > **T**	**A03**	**14295938**	**0.00**	**4.13**	**2.46**	**0.62**
	3077572	A05	2448983	0.00	4.97	3.08	−0.78
	3120357_8:G > T	C04	16700301	0.00	3.90	2.29	−0.69
	3134332	C06	956033	0.00	4.03	2.45	−0.72
	4116149_16:G > A	C08	36631450	0.00	3.92	2.36	0.66
Field evaluation in disease nursery (Green Lake)-site III	3147873_8:C > T	A10	526147	0.00	4.35	4.96	−1.09
	5034116_49:T > G	A10	14099074	0.00	3.73	3.95	−0.62
Ascospore shower test with pathotypes from mixed stubble	**3196349_56:A** > **C**	**A01**	**4839375 approx**	**0.00**	**4.63**	**12.56**	−**0.85**
	**100027433_25:T** > **C**	**A03**	**13164795**	**0.00**	**2.31**	**5.88**	**0.51**
	3121801_61:A > T	A04		0.00	3.94	10.84	0.63
	5756681_34:A > T	A07	19373605	0.00	3.09	8.20	0.58
	3087797_27:C > G	C03	2032503	0.00	4.15	11.44	−0.92
	3133149_28:G > A	C04	*30423579	0.00	3.70	9.93	−0.74
Cotyledon test with IBCN17 isolate	**3076956_16:G** > **A**	**A07**	**15907056**	**0.00**	**77.37**	**81.88**	**0.68**
	3099202_14:C > A	A09	15423340	0.00	3.17	8.25	0.09
Cotyledon test with IBCN76 isolate	**100027433_25:T** > **C**	**A03**	**13164795**	**0.01**	**2.26**	**4.73**	**0.06**
	**3076956_16:G** > **A**	**A07**	**15907056**	**0.00**	**50.54**	**63.44**	−**0.59**
	100030684_5:C > T	C02	3442359	0.00	3.02	6.66	−0.08
	3104923_39:A > T	C03	3559949	0.00	3.73	9.21	0.13
	3090406_36:C > G	C05	36183396	0.00	3.68	8.11	0.09

Associations in bold represent to those which appeared at least in two experiments.

**Table 3:** Whole Genome Average Interval mapping approach based QTL identified for resistance to *L. maculans* under glasshouse and field conditions in a doubled haploid population derived from RP04/Ag-Outback.

	Chromosome	Left Marker	Genetic Distance (cM)	Right Marker	Genetic Distance(cM)	QTL Size	P value	Genetic variance (%)	LOD
Ascospore shower test with pathotypes from mixed stubble	A01	4108895_33:A > G	87.71	3196349_56:A > C	88.3	−1.03	<0.001	12.4	9.36
Field evaluation in nursery at Wagga Wagga (2013) - Site I	A01	3094707_39:C > A	98.89	5031260_16:C > A	100.06	−0.27	<0.001	10.2	6.17
Ascospore shower test with pathotypes from mixed stubble	A03	100027433_25:T > C	1.81	3098300_24:G > T	2.39	1.24	<0.001	18.1	8.17
Field evaluation in nursery at Wagga Wagga (2013) - Site I	**A03**	**5238867_9:G** > **T**	**14.16**	**4121772**	**14.75**	**0.17**	**<0.001**	**3.8**	**3.04**
Wagga disease nursery irrigated - Site II	**A03**	**5238867_9:G** > **T**	**14.16**	**4121772**	**14.75**	**0.76**	**<0.001**	**23.6**	**5.80**
Ascospore shower test with pathotypes from mixed stubble	A03	3115947_31:A > T	21.81	5708870	25.33	−0.75	<0.001	6.6	3.02
Field evaluation in nursery at Wagga Wagga (2013) - Site I	A05	3155734_44:A > G	21.56	3142329_10:A > G	22.15	−0.19	<0.001	4.9	3.18
Field evaluation in nursery at Wagga Wagga (2013) - Site I	A06	5047705_5:T > C	80.08	3118601_36:G > A	80.67	0.22	<0.001	6.9	5.54
Field evaluation in nursery at Wagga Wagga (2013) - Site I	A07	5030824_10:T > C	20.59	3113568_47:G > A	22.35	0.22	<0.001	6.6	4.35
Cotyledon test with IBCN17 isolate	**A07**	**3076956_16:G** > **A**	**67.06**	**3096079_47:A** > **T**	**68.82**	**0.68**	**<0.001**	**90.1**	**90.00**
Cotyledon test with IBCN76 isolate	**A07**	**3076956_16:G** > **A**	**67.06**	**3096079_47:A** > **T**	**68.82**	−**0.58**	**<0.001**	**80.8**	**97.76**
Ascospore shower test with pathotypes from mixed stubble	**A07**	**3096079_47:A** > **T**	**68.82**	**3085294_14:A** > **C**	**69.41**	**0.49**	**<0.001**	**2.8**	**2.65**
Ascospore shower test with pathotypes from mixed stubble	A09	3113689_32:T > C	100.14	4120699_33:A > G	100.73	0.61	<0.001	4.4	3.56
Field evaluation in nursery at Green Lake - Site III	A10	3147873_8:C > T	29.42	4121514	30.01	−0.65	<0.001	33.3	3.02
Ascospore shower test with pathotypes from mixed stubble	A10	100030281_38:A > G	8.82	4120120	9.41	0.75	<0.001	6.7	5.16
Field evaluation in nursery at Wagga Wagga (2013) - Site I	A10	4113344_44:C > T	24.71	3094654_53:A > C	25.29	0.21	<0.001	5.9	4.36
Field evaluation in nursery at Wagga Wagga (2013) - Site I	C01	5052279	3.56	100030685_9:G > T	4.15	−0.16	<0.001	3.4	2.85
Ascospore shower test with pathotypes from mixed stubble	C02	4118276	120.67	3124480_13:C > T	122.44	−0.81	<0.001	7.7	5.73
Field evaluation in nursery at Wagga Wagga (2013) - Site I	C03	3080832	6.49	3087797_27:C > G	14.78	−0.27	<0.001	10.1	2.70
Ascospore shower test with pathotypes from mixed stubble	C03	7247685	47.72	4168594_22:T > C	48.31	−0.65	<0.001	5	3.45
Field evaluation in nursery at Wagga Wagga (2013) - Site I	**C04**	**4168434_28:G** > **A**	**0.59**	**3150569_22:A** > **C**	**1.18**	**0.31**	**<0.001**	**13.1**	**5.95**
Wagga disease nursery irrigated - Site II	**C04**	**4168434_28:G** > **A**	**0.59**	**3150569_22:A** > **C**	**1.18**	**0.66**	**<0.001**	**18**	**3.05**
Ascospore shower test with pathotypes from mixed stubble	C04	3086475_43:T > C	49.41	3133149_28:G > A	50.59	−0.93	<0.001	10.3	7.67
Field evaluation in nursery at Wagga Wagga (2013) - Site I	C07	100042985_9:A > G	24.15	5152626	24.74	0.17	<0.001	4.2	3.27
Cotyledon test with IBCN76 isolate	C09	5154774	2.41	3082399_21:A > G	3	−0.11	<0.001	2.8	3.13

#### Genetic mapping for field resistance

Three experiments were conducted to assess resistance to *L. maculans;* two at Wagga Wagga and one at Green Lake. Up to 11 significant marker associations were identified using both genome scan and WGAIM approaches on chromosomes A01, A03, A05, A06, A07, A10, C04, C06 and C08 in the RADH population (Tables 2 and 3). Interestingly, two QTL on chromosomes A03 and C04 were repeatedly detected across experiments, suggesting that environment; including prevalence of diverse races of *L. maculans* may have contributed toward inconsistent detection of QTLs. The percentage of genotypic variation accounted by individual SNP markers ranged from 3.4% (C01) to 33.3% (A10, Table 3). Among the significant SNPs, 3147873_8:C > T/4121514 marker interval explained the maximum genetic variation (33.3%) for resistance on chromosome A10.

#### Genetic mapping for resistance with ascospore shower test

Nine marker intervals showed significant associations for resistance to mixed *L. maculans* pathotypes originated from stubble sources, on chromosomes: A01, A03, A07, A09, A10, C2 C03, and C04 (Table 3). The genotypic variation explained by these QTL ranged from 2.8% to 18.1%. DArTseq SNP marker interval 4108895_33:A > G/3196349_56:A > C showed highly significant association (LOD = 9.36) on chromosome A01 and accounted for 12.4% of genetic variation in resistance to *L. maculans*, followed by marker interval 100027433_25:T > C/3098300_24:G > T with LOD score of 8.17 on chromosome A01, which accounted for 18.1% of genetic variation (Table 3). Collectively these QTL accounted for 48.76% of QR in RADH population. Both parents contributed favorable alleles for QR.

### Mapping of race-specific resistance to *L. maculans*

**Figure 3:**
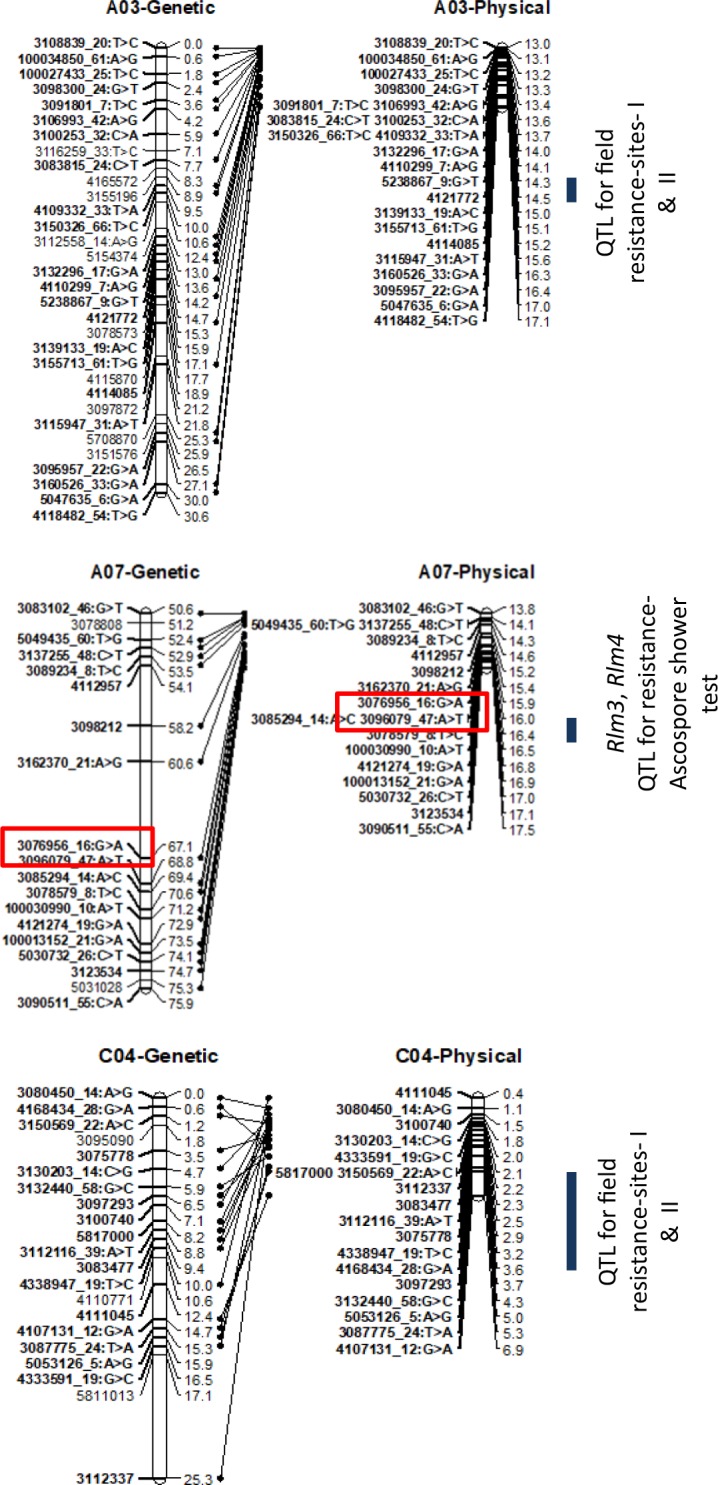
Partial genetic linkage maps of chromosomes A03, A07 and C04 of *B. napus* DH population from RP04/Ag-Outback, showing localization of *Rlm3, Rlm4* genes (in rectangle) as well as QTL for resistance to *L. maculans*. Physical map positions of significant associations are shown on the reference sequence of *B. napus v4.1*.

Six significant associations were detected for resistance to SSIs of *L. maculans* using genome scan approach; two SNP associations were detected with IBCN17 isolate on chromosomes A07 and A09, while four were detected with isolate IBCN76 on chromosomes A07, C02, C03 and C05 (Table 2). WGAIM detected two QTL regions for resistance to SSIs on chromosomes A07 and C09 (Table 3). The genotypic variation explained by individual SNP ranged from 2.8% to 90.1%. Highly significant marker interval 3076956-16:G > A/3096079_47:A > T (LOD = 90.0) on chromosome A07 accounted for 90.1% of the genetic variation for resistance to isolate IBCN17. An additive effect was also contributed by the maternal parent, RP04 (Tables 2 and 3). Several markers (20) showed complete segregation with DArTseq SNP marker 3076956-16:G > A on A07 (Supplementary Table 2), suggesting that these markers can be used for selection of resistance in different genetic backgrounds. The same marker interval; 3076956_16:G > A/3096079_47:A > T also showed significant association (LOD = 97.76) for resistance to isolate IBCN76, accounting for up to 80.8% of the genetic variation (Table 3, Fig. 3). However, an additive effect was also contributed by the paternal parent, Ag-Outback. This differential avirulent response of Ag-Outback to isolate IBCN76 confirmed the similar allelic effect revealed by the QTL mapping approaches. Given that SNP markers (3076956_16:G > A and 3096079_47:A > T on A07) accounted for a large proportion of genotypic variation for resistance to *L. maculans* (Table 3, Fig. 3), the disease scores from cotyledon tests were binned into two discrete categories; lines having average scores of ≤3 were classified as resistant and lines that have ≥4 were rated as susceptible. Linkage analysis revealed that a major locus for resistance to *L. maculans*; *Rlm3* is located on chromosome A07 (Table 3). Linkage analysis between phenotypic binned data from cotyledon tests and markers present on a physical map of chromosome A07 of the reference genome showed that both the *Rlm3* and *Rlm4* genes are located on chromosome A07 (Fig. 3). The location of both *R* genes determined here is consistent with the previous mapping studies conducted on populations from Maxol/Westar, Columbus/Westar, Ag-Castle/Westar, and Ag-Castle/Topas^3,5,10^.

### Consistent QTL detected for resistance

Of the 19 significant QTLs identified with WGAIM, the allelic effects of the A03, A07 and C04 loci were detected repeatedly at least in two phenotypic environments (Table 3). The genetic effect of the A03 and C04 loci was repeatedly detected under field conditions at Wagga Wagga sites. Other consistent QTL on chromosome A07 for resistance to *L. maculans* evaluated by ascospore shower test was localized within the marker-intervals for *Rlm3* and *Rlm4*. QTL for field resistance on chromosome A01 was also identified within 1 Mb of previously identified QTL for QR in *B. napus* populations^5,35^.

### Identification of putative candidate resistance genes

To obtain the physical positions of putative *R* genes, 19 DArTseq SNP markers that showed significant association (LOD ≥ 3) with *R* and QR loci in the RADH population (Table 4), we searched for their identities against the reference sequence of Darmor using BLASTN. Only 60% of marker sequences were mapped on the reference sequence (Supplementary Table 1), suggesting that some of the genomic variation in *B. napus* varieties (e.g. RP04 and Ag-Outback) is not represented in the reference Darmor genome. Several markers were mapped on ChrAnn_ran contigs which have not been anchored on the reference *B. napus*.

**Table 4:** Candidate gene located in the vicinity of significant associations linked with qualitative and quantitative resistance loci to *L. maculans*. Only candidates that mapped within 10 kb from significant trait-marker associations are shown herein.

SNP marker	Physical location of SNP on Darmor sequence	Location of candidate gene near SNP (predicted *B. napus* gene and physical position (bp) in *B. napus* genome	Marker distance from the nearest predicted gene in *B. napus* genome (bp)	Annotated gene in the *A. thaliana* genome (Gene ID)	BLASTp alignment score (Score bits/E value)	Gene Function
100027433_25:T > C	13,164,795 (A03)	BnaA03g26760D: 13160577 to 13167611	2,186	Transcription factor jumonji (AT4G00990.1)	669/0.0	Epigenetic regulation of defense response
5238867_9:G > T	14290571(A03)	BnaA03g29380D 14293489 to 14296428	2819	Zinc finger, RING/FYVE/PHD-type (AT3G06330.1))	762/0.0	RING/U-box superfamily protein
3076956_16:G > A	15,928,008 (A07)	BnaA07g20240D: 15920100 to 15923891	6,883	Protein kinase superfamily protein (AT1G79640.1)	1,234/0.0	Protein serine/threonine kinase activity, protein kinase activity, kinase activity, ATP binding
4168434_28:G > A	~1831733 (C04)	BnaC04g02370D: 1828557 to 1831604	129	Tetratricopeptide repeat	1790/0.0	TPR repeat-containing thioredoxin TTL3

Physical mapping of markers linked with consistent resistance loci on the ‘Darmor’ genome assembly enabled identification of three candidate genes: BnaA03g26760D (A03), BnaA07g20240D (A07), and BnaC03g04240D (C03) (Table 4). These genes were searched for their identities against *A. thaliana* genome (TAIR 10 protein database). The BnaA03g26760D gene showed significant sequence identities (669 bits, E value = 0) with Transcription factor *JUMONJI* (AT4G00990.1), while the BnaA07g20240D (A07) gene showed sequence identities with Protein kinase superfamily protein of *A. thaliana* (AT1G79640.1; score bits: 1234, e value: 0.0). This gene has been previously mapped close to the *Rlm4* locus for blackleg resistance^36^. BnaC03g04240D (C03) showed sequence similarities with AT5G10370.1 gene encoding helicase domain-containing protein (score: 2806, e value: 0.0). *JUMONJI* has been shown specifically regulated in the resistant SA1306 genotype suggesting that it act as major regulators of transcription defense responses against *Erysiphe pisi* in *Medicago*^37^. *JUMONJI C* has also been implicated in defense response against *Xanthomomas oryzae pv. oryzae* infection^38^.

## Discussion

Virulent strains of *L. maculans* constantly threaten the sustainability of canola industry worldwide. Therefore, identification and deployment of both effective *R* and QR genes is needed for stable canola production. In previous studies, genetic resistance to *L. maculans* has been mapped in various Australian populations derived from Ag-Castle, AG-Spectrum, AV-Sapphire, Dunkeld, Maluka, Rainbow, Skipton, Shiralee and Surpass400^3–6,8–10,39–41^. Both parental lines of the RADH population, RP04 and Ag-Outback, have shown good levels of blackleg resistance under field conditions previously. In this study, we investigated the genetic basis of resistance and identified QTL for resistance to *L. maculans* in RADH population across six phenotypic environments. Among 11 significant SNP associations for field resistance, only two of them were detected repeatedly across experiments in both 2013 and 2015. This suggested that detection of QR (multi-genic) loci that are effective in conferring resistance to *L. maculans* across diverse environments is a challenging exercise. Highly heterogeneous populations of this pathogen^42^ may have contributed towards inconsistent detection of significant associations across environments (Tables 2 and 3). Secondly, experiments were conducted in different agro-climatic conditions which may have contributed to the different level of disease expression. For example in Lake Green, disease scores of DH lines had very low repeatability (12%), although canker scores were high (Table 1). In order to reduce the environmental variation and to increase selection efficiency for resistance, we utilized the ‘ascospore shower’ test to identify disease resistance loci under shade-house conditions. At least one QTL on chromosome A07 corresponding to the RP04 was detected with IBCN17 and ascospore shower test. This suggested that ascospore test is suitable for detecting both R and QR genes for resistance to *L. maculans*. Our results show that heritability increased from 12% (field experiment-site III) to 61% (under shade-house conditions, Table 1), suggesting that the ascospore shower can be reliably used for identification of QTL associated with resistance to blackleg pathotypes. In addition, a highly significant genetic association for resistance to *L. maculans* pathotypes was identified on chromosome A01 in RADH population. In previous studies, significant associations for resistance on A01 were reported in DH populations from Skipton/Ag-Spectrum, Topas/Ag-Castle and Topas/AV-Sapphire and diverse panels of *B. napus*^3,5,10,17^. Our results have also shown that race-specific major genes; *Rlm3* and *Rlm4* are ineffective at conferring resistance to *L. maculans* in RADH under Australian field conditions, as no QTL in the vicinity of *Rlm3*/*Rlm4* genomic regions was identified on chromosome A07 (Table 4). It is probable that *Rlm3-* and *Rlm4*-attacking isolates were present in the phenotyping environments used, rendering both *R* genes ineffective. Our results are consistent with a previous study which shows that *Rlm3* and *Rlm4* are not very effective in providing resistance to *L. maculans* under Australian field conditions^22^. We identified 4 to 6 QTL associated with resistance using SSI (Table 3). While A07 loci represents to *Rlm3* and *Rlm4* genes, the role of other QTL on chromosomes A09, C2, C3, and C5 in conferring resistance to *L. maculans* need to be established. Since single spore isolates have multiple avirulence genes, it is possible that these loci may either (i) represent specific specificities, consistent with the gene-for-gene interaction (Flor 1971) in the *B. napus-L. maculans* pathosystem, or (ii) are genes triggered in response to pathogen attack (signaling and transduction). In *B. napus*, QR to *L. maculans* has been mapped onto chromosomes A09, A10, C02, C03 and C05^3,5,10^.

In conclusion, we found that two race-specific genes *Rlm3*, *Rlm4* and 19 significant QTLs control resistance to *L. maculan* in the DH population derived from the RP04/Ag-Outback cross. However, many stable QTLs could not be detected across environments as the QTL detection relies heavily on the phenotyping environment. QTL identified across multiple environments (years/sites) can be incorporated for enhancing effective QR in canola breeding germplasm. Although, the majority of QTL had small genetic effects, the genomic selection strategy could be used to combine a large number of environment-specific and environment non-specific (stable) QTL to develop varieties with durable resistance. Our study also showed that both *Rlm3* and *Rlm4* genes are ineffective in providing resistance under Australian field conditions. Therefore other strategies such as rotation of race-specific genes as well as deployment of stable quantitative resistance are required for sustainable blackleg management and canola production. Overall our results provide an insight into the *R* and *QR* loci for resistance to *L. maculans* in RP04/Ag-Outback.

## Supplementary information

Supplementary Information.

Supplementary Table S2.
