# Supplementary material for: Genetic and physical mapping of loci for resistance to blackleg disease in canola (Brassica napus L.)
Source: Sci Rep. 2020 Mar 10;10:4416. doi: 10.1038/s41598-020-61211-y (PMC7064481; doi:10.1038/s41598-020-61211-y)
Supplement: Supplementary file 1 — Supplementary Information. [file 41598_2020_61211_MOESM1_ESM.pdf]

Supplementary Information

Genetic and physical mapping of loci for resistance to blackleg disease in canola (*Brassica napus* L.)

Rosy Raman[1], Simon Diffey[2], Denise M. Barbulescu[3], Neil Coombes[1], David Luckett1,[1,4], Phil Salisbury[3,5], Raymond Cowley[1, 6], Steve Marcroft[7] and Harsh Raman[1. 4*]

[1]NSW Department of Primary Industries, Wagga Wagga Agricultural Institute, Wagga Wagga, NSW 2650, Australia. [2]Apex Biometry, South Freemantle, WA 6162, Australia.[3]Agriculture Victoria, Grains Innovation Park, Horsham 3401, VIC, Australia; [4]Graham Centre for Agricultural Innovation (a joint venture between Charles Sturt University and NSW Department of Primary Industries), Charles Sturt University, Boorooma Street, Wagga Wagga NSW 2650. [5]Faculty of Veterinary and Agricultural Sciences, The University of Melbourne, Vic 3010, Australia. [6] Present address: Corteva Agrosciences, Lawson Street, Wagga Wagga, NSW 2650. [7] Marcroft Grains Pathology, Grains Innovation Park, Horsham, Victoria 3400, Australia

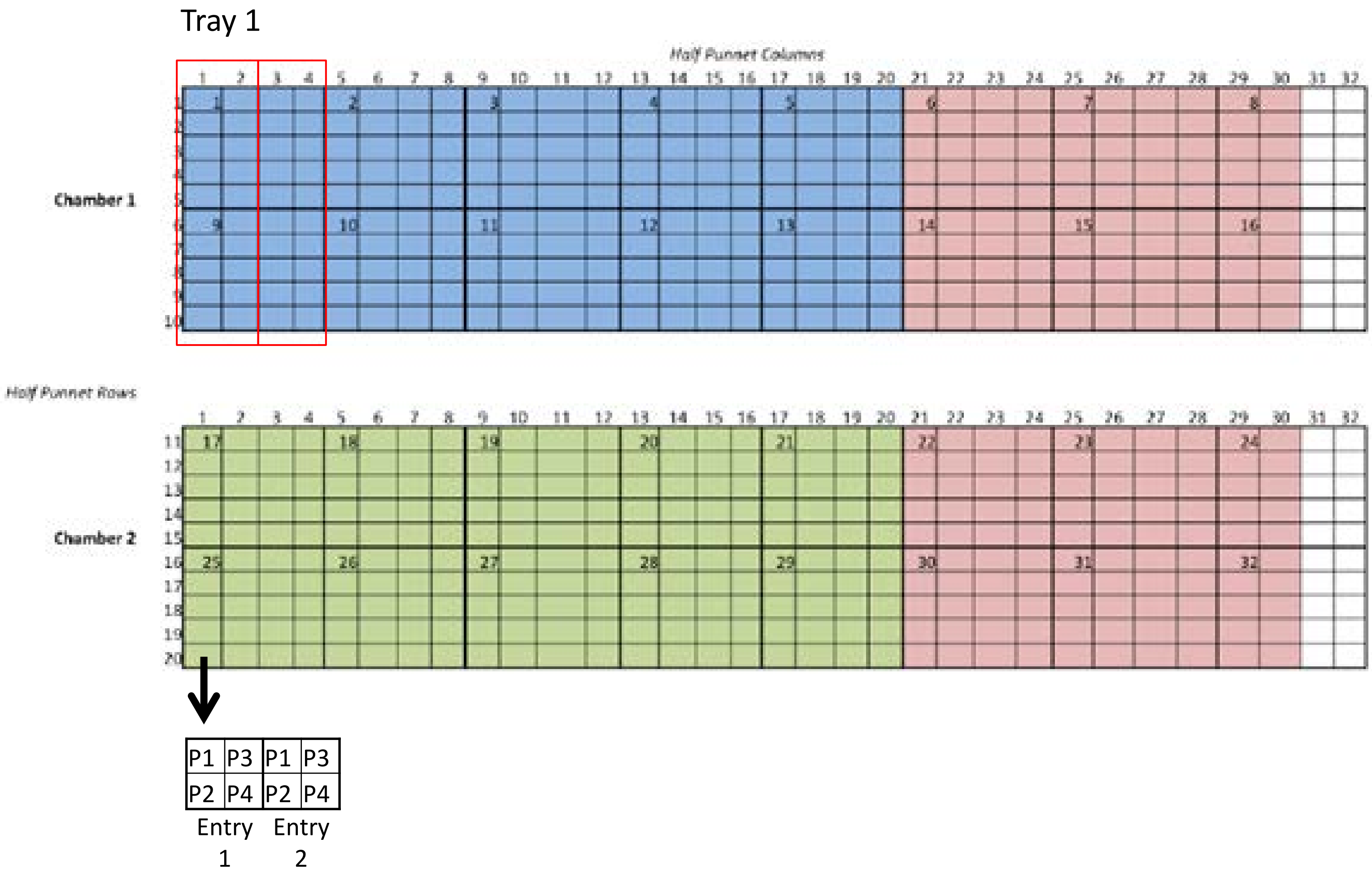

Supplementary Figure 1: Experimental layout-Plants were grown in punnets; each punnet comprised 8 cells and accommodate two entries. A total of 10 punnets/tray was placed on bench as shown above. A total of 15 trays were used per bench to evaluate 300 entries per chamber. This design enabled to evaluate 1.5 replicates of 171 RADH lines, two entries of each parent of the RADH population plus 13 entries of Skipton and 12 entries of Ag-Spectrum. Two chambers were used to evaluate 3 replicates of 200 entries. Experiment was conducted at Marcroft Grains Pathology, Horsham, Australia

Supplementary Table 1: Differential response of accessions (hosts) to the single spore isolates of L. maculans causing blackleg disease in canola. Avirulence response was assessed according to Marcroft et al (2012)											
Experiment 1a											
Genotype	D1	D2	D3	D4 (IBCN17)	D5	D7 (IBCN76)	D8	D9	D10	D13	Predcited R gene
RP004	2.62	4.32	3.44	2.64	2.64	5.53	5.75	4.94	6.91	1.71	*Rlm4*
Ag-Outback	4.07	3.63	-	5.81	6.78	1.00	4.84	6.38	5.67	5.44	*Rlm3*
Mustang	1.63	1.00	5.69	1.00	1.75	1.06	4.13	1.25	1.50	1.00	*Rlm6*
Q2	2.73	5.75	5.69	6.63	6.69	1.44	6.00	6.13	4.81	5.44	*Rlm3 ,9?*
Surpass 400	1.13	1.65	4.35	3.06	1.00	1.63	7.34	5.78	2.00	4.81	*Rlm1, LepR3*
Thunder	1.91	6.16	4.00	3.34	1.56	5.08	4.06	4.31	3.94	1.25	*Rlm4*
Westar	3.22	7.69	5.44	6.38	6.27	6.09	6.13	6.53	5.94	6.06	*Susceptible*
Caiman	4.50	7.22	5.38	1.29	1.13	4.66	1.31	1.19	4.97	1.25	*Rlm7*
Garnet	1.88	5.94	6.88	6.06	1.81	1.44	5.94	4.75	3.00	5.13	*Rlm1, 9*

Experiment 1b. Screening of parental lines and their DH progenies for resistance to IBCN17 and IBCN76 isolates of L. maculans. Additional eight lines were used as internal controls.			
Genotype	IBCN17	IBCN76	
RP004	2.62	5.65	*Rlm4*
AG-OUTBACK	5.25	2.66	*Rlm3*
Mustang	2.07	5.63	
Q2	5.31	2.38	
Surpass400	4.42	2.01	
Thunder	2.47	5.51	
Westar	5.49	5.48	
Garnet	5.28	2.36	
44C79CL	5.47	4.92	
DH means (RP04/	3.94	4.75	
Hyola50	2.08	1.70	

Supplementary Table 3. Summary of markers, and their distribution and density across different linkage groups and subgenomes (A_n and C_n) of the RP04/Ag-Outback DH population of *B. napus.*

	Total mapped markers	Number of bin marker loci	Genetic map length	Average bin markers /cM
An01	1137	200	260.66	0.77
An02	813	75	95.78	0.78
An03	1404	66	76.69	0.86
An04	513	78	83.29	0.94
An05	1253	140	179.43	0.78
An06	761	74	96.67	0.76
An07	907	96	121.76	0.79
An08	149	23	23.73	0.97
An09	1528	180	314.28	0.57
An10	956	119	122.37	0.97
Subtotal of the An	***9421***	**1051**	**1374.66**	***0.76***
Cn01	1244	97	107.72	0.90
Cn02	1452	165	227.21	0.73
Cn03	1106	133	226.55	0.59
Cn04	1942	112	180.28	0.62
Cn05	449	80	122.95	0.65
Cn06	803	125	83.57	1.50
Cn07	1071	131	147.71	0.89
Cn08	923	84	88.445	0.95
Cn09	440	41	57.65	0.72
Subtotal of the Cn	***9430***	***968***	***1242.08***	***0.78***
**Total of the AnCn**	**18851**	**2019**	**2616.74**	**0.77**

Supplementary Table 4: Statistical association between DArTseq SNP markers and resistance to *L. maculans* in a doubled haploid population derived from the RP04/Ag-outback. Associations in bold represent to those which appeared at least in two experiments. QTL with LOD ≥3 were assigned as ‘significant’ while QTL with ≤ 2.9 were assigned as ‘suggestive’.

Phenotyping environment	DArTseq marker	Chromosome	Physical position of marker on the Darmor sequence	Probability of association	LOD Score	Genotypic variation explained (%)	Allelic effect
Field evaluation in disease nursery (Wagga Wagga)-site 1	**5031260_16:C>A**	**A01**	**6158899**	**0.00**	**4.16**	**5.39**	**-0.29**
	5029602_37:T>C	A02	5780342	0.00	2.76	6.66	-0.18
	3155734_44:A>G	A05	*3225148	0.00	2.35	2.44	-0.20
	3087048_29:C>T	A05	22618486	0.01	2.15	3.17	0.15
	3118601_36:G>A	A06	20027453	0.00	3.16	4.83	0.20
	3113568_47:G>A	A07	10654960	0.00	2.60	3.54	0.20
	100032109_46:A>C	A07	*22062589	0.01	2.06	3.59	-0.16
	3126064_15:G>C	A07	*20050262	0.01	2.07	5.29	0.17
	3091258_29:G>A	A10	11581532	0.00	3.18	5.14	0.22
	3087797_27:C>G	C03	2032503	0.01	1.99	3.93	-0.19
	4168434_28:G>A	C04	3595356	0.01	2.10	3.16	0.20
	5756848	C04	*36924163	0.01	2.12	3.26	-0.16
Field evaluation in disease nursery (Wagga Wagga)-site II	**5238867_9:G>T**	**A03**	**14295938**	**0.00**	**4.13**	**2.46**	**0.62**
	3077572	A05	2448983	0.00	4.97	3.08	-0.78
	4110716_58:A>C	A05	*5390657	0.01	2.29	1.25	0.50
	3120357_8:G>T	C04	16700301	0.00	3.90	2.29	-0.69
	3134332	C06	956033	0.00	4.03	2.45	-0.72
	4332800	C06	6283199	0.00	2.75	1.55	-0.55
	3151563_46:C>T	C06	34266792	0.00	2.98	1.70	-0.50
	4116149_16:G>A	C08	36631450	0.00	3.92	2.36	0.66

	3111823_16:T>C	A09	29380072	0.01	1.86	1.81	0.39
	3159803_10:T>C	A09	30918459	0.01	2.03	1.99	0.71
	3147873_8:C>T	A10	526147	0.00	4.35	4.96	-1.09
	5034116_49:T>G	A10	14099074	0.00	3.73	3.95	-0.62
Field evaluation in disease nursery (Green Lake)-site III	3088593	C02	45436071	0.00	2.48	2.50	0.49
Ascospore shower test with pathotypes from mixed stubble	**3196349_56:A>C**	**A01**	**4839375 approx.**	**0.00**	**4.63**	**12.56**	**-0.85**
	**100027433_25:T>C**	**A03**	**13164795**	**0.00**	**2.31**	**5.88**	**0.51**
	3121801_61:A>T	A04		0.00	3.94	10.84	0.63
	5756681_34:A>T	A07	19373605	0.00	3.09	8.20	0.58
	3095003	Ann_random	593366	0.01	2.15	5.42	0.48
	4118276	C02	*19842374	0.00	2.35	5.99	-0.55
	3087797_27:C>G	C03	2032503	0.00	4.15	11.44	-0.92
	3133149_28:G>A	C04	*30423579	0.00	3.70	9.93	-0.74
Cotyledon test with IBCN17 isolate	4116509	A02	3783124	0.00	2.41	5.44	0.68
	4115870	A03	15066837	0.01	2.02	3.82	0.09
	**3076956_16:G>A**	**A07**	**15907056**	**0.00**	**77.37**	**81.88**	**0.68**
	3099202_14:C>A	A09	15423340	0.00	3.17	8.25	0.09
Cotyledon test with IBCN76 isolate	**100027433_25:T>C**	**A03**	**13164795**	**0.01**	**2.26**	**4.73**	**0.06**
	**3076956_16:G>A**	**A07**	**15907056**	**0.00**	**50.54**	**63.44**	**-0.59**
	5756923_14:G>C	A10	5266121	0.00	2.79	6.07	0.07
	100030684_5:C>T	C02	3442359	0.00	3.02	6.66	-0.08
	3104923_39:A>T	C03	3559949	0.00	3.73	9.21	0.13
	3090406_36:C>G	C05	36183396	0.00	3.68	8.11	0.09
